# Surface Molecularly Imprinted Polymer Film with Poly(*p*-aminothiophenol) Outer Layer Coated on Gold Nanoparticles Inner Layer for Highly Sensitive and Selective Sensing Paraoxon

**DOI:** 10.3390/polym9080359

**Published:** 2017-08-12

**Authors:** Shanshan Li, Qingying Luo, Yaowen Liu, Zhiqing Zhang, Guanghui Shen, Hejun Wu, Anjun Chen, Xingyan Liu, Aidong Zhang

**Affiliations:** 1 College of Food Science, Sichuan Agricultural University, Ya’an 625014, China; cherry12112009@163.com (Q.L.); yl038024097@126.com (Y.L.); zqzhang721@163.com (Z.Z.); shenghuishen@163.com (G.S.); hejunwu520@163.com (H.W.); anjunc003@163.com (A.C.); lxy05@126.com (X.L.); 2 Key Laboratory of Pesticide & Chemical Biology of Ministry of Education, College of Chemistry, Central China Normal University, Wuhan 430079, China

**Keywords:** molecularly imprinted polymer, surface molecularly imprinting, self-assembly directing, paraoxon, gold nanoparticles, electro-polymerization, electrochemical sensor

## Abstract

This paper presents the fabrication of a molecularly imprinted, polymer-based disposable electrochemical sensor for paraoxon (PO) determination. The sensor was based on a screen-printed carbon electrode (SPCE) modified with a surface molecularly imprinted poly (*p*-aminothiophenol) (PATP)/gold nanoparticles (AuNPs) composite film, which consisted of a PATP outer layer and an AuNPs inner layer. We report a novel strategy, combining surface molecularly imprinting and self-assembly directed electro-polymerization with high densely imprinting PO molecules in the PATP/AuNPs film. Firstly, AuNPs were in situ electrodeposited at the electrode surface, and then assembled with electropolmerizable functional monomer *p*-aminothiophenol (ATP). Subsequently, PO molecules were assembled onto the ATP monolayer-modified AuNPs, forming a basis of surface molecular imprinting. After that, replenished PO molecules were embedded in the PATP/AuNPs film by PO and the ATP molecular self-assembly directed electro-polymerization in the polymerization precursor mixture. The resulting imprinted PATP/AuNPs/SPCE possesses high sensitivity, affinity, and selectivity toward PO, with a low detection limit of 1 × 10^−9^ M. The proposed sensor was successfully applied for the determination of PO in fruit and vegetables, giving satisfactory recoveries. The strategy reported herein can be further expected to fabricate various molecular imprinted sensors for the determination of other pesticide residuals.

## 1. Introduction

Highly neurotoxic organophosphates, such as paraoxon (PO), are widely used as persistent pesticides and nerve agents, and are responsible for a number of poisonings. Organophosphate can disrupt the cholinesterase enzyme that catalyzes the breakdown of acetylcholine, a neurotransmitter needed for proper nervous system function, which often causes convulsions, coma, respiratory paralysis, and death [1,2]. In view of the their high toxicity, developing simple, fast, and sensitive methods for the specific determination of organophosphates is of great interest in environmental, food safety, and human health concerns [3,4,5]. Different analytical methods have been used to detect organophosphates in the past decade, including liquid or gas chromatography, often coupled to mass spectrometry [6,7] and biological methods, such as immunoassay [8] and enzyme catalysis of organophosphates hydrolysis [9], or inhibition of cholinesterase activity [10]. Although sensitive, these different analytical protocols suffer from time-consuming sample pretreatments, poor specificity, long analysis time intervals, complex analytical protocols, expensive instruments, and, moreover, potable and fast online tests are impractical.

As an alternative approach—molecularly imprinted polymers (MIPs) that exhibit high selectivity and affinity to the predetermined specific species—are now experiencing fast research growth, and have been applied as the sensor recognition element for organophosphates determination [11,12,13]. The molecularly imprinting technique is based on the preassembly of the template molecule and the functional monomer, and is subsequently copolymerized with cross-linking monomers. The removal of the template leaves selective recognition sites that are complemented to the target analyte in the imprinted substrates. This artificial molecular recognition system has significant advantages such as mechanical/chemical stability, low cost, and ease of preparation, thus have increasingly attracted considerable attentions [14,15]. However, the use of conventional MIPs as functional sensing matrices suffers several basic limitations, including incomplete template removal, small binding capacity, poor site accessibility to target species, and inefficient communication between the binding sites and the transducers [16]. For one thing, it is quite difficult to remove templates from the inside of the high cross-linked bulk imprinted polymers [17], which reduce the capacity of the rebinding target analyte. For another, if the generated template-shaped cavities are not at the surface or in close proximity to the materials’ surface, the high mass transfer resistance will hinder the targets from accessing the inner imprinted cavities, resulting in poor site accessibility in the imprinted polymers [18].

Addressing the limitations of conventional molecular imprinting techniques, several research groups have begun to explore alternative approaches for developing new imprinting methodologies. An effective approach is to control templates to locate at the surface of imprinted materials, typically exampled by surface imprinting, which is carried out by immobilizing template molecules at the surface of suitable substrates, forming thin imprinted films [19,20]. Surface molecularly imprinting is especially valuable as it solves the problems of limited mass transfer and template removal to some extent, as compared to the conventional molecularly imprinting technique. Another attempt to address the limitations of the conventional MIPs is the development of molecularly imprinted nanomaterials [21]. Nanostructured imprinted substrates have extremely high surface-to-volume ratio, so that most of the template molecules can be situated at the surface of the materials, resulting in a large amount of effective imprinted sites, a high binding capacity, and good site accessibility for the molecularly imprinted nanomaterials [22,23]. In view of the advantages of the two approaches, we wish to incorporate both of them to develop surface molecularly imprinted nanomaterials for the sensitive and selective analysis of organophosphates. 

**Scheme 1:**
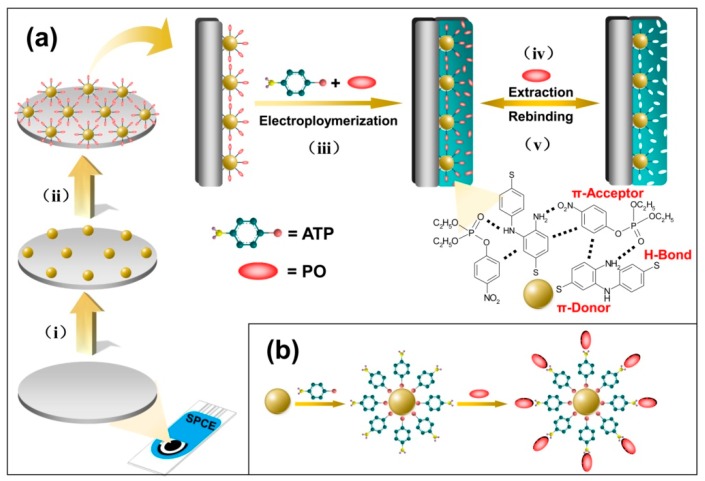
(**a**) Preparation procedures of the imprinted *p*-aminothiophenol (PATP)/gold nanoparticles (AuNPs)/screen-printed carbon electrode (SPCE) and the principle for recognition of paraoxon (PO): (i) electrodeposition of an AuNPs inner layer on the surface of the SPCE; (ii) ATP assembled onto the AuNPs and following PO assembled onto the formed ATP–AuNPs, forming a basis of surface molecular imprinting; (iii) electropolymerization of PATP film on the surface of the electrode in the presence of PO molecules in the polymerization precursor mixture; (iv)/(v) removal/rebinding of PO on the imprinted sites of the imprinted PATP/AuNPs/SPCE; (**b**) schematic illustrations for the adsorption of the ATP molecules at the AuNPs surface and the further self-assembly of PO at the ATP–AuNPs.

Herein, we fabricated a novel disposable electrochemical sensor based on surface molecularly imprinted PATP/AuNPs composite film for sensitive and selective analysis of PO. Specially, we demonstrated a self-assembly directing strategy for the highly dense imprinting of the template molecules in the electropolymerized PATP/AuNPs film at the disposable screen printed carbon electrode (SPCE) surface (see Scheme 1). For the sensor construction, AuNPs were electrodeposited onto the SPCE surface and then were modified with the electropolymerizable functional monomer ATP via molecular self-assembly. This ATP (self-assembled, monolayer, (SAM)-modified AuNPs (ATP–AuNPs)) can serve as a high-conductive, nanostructured, imprinted inner layer. The initial PO imprinted sites were generated by the surface imprinting of the PO molecules to the ATP–AuNPs, and then the imprinted sites were further replenished by the subsequent electropolymerization of the PATP film in the presence of PO moleculars in the polymerization precursor mixture. It was expected that the ATP SAM on the AuNPs would drive PO molecules to richen onto the electrode surface to form high dense imprinted sites, and simultaneously direct the selective occurrence of the imprinting electropolymerization of ATP at the electrode, forming a high-quality uniform MIP film [24]. Thus, the developed surface molecularly imprinted PATP/AuNPs composite film would concentrate PO with high efficiency at the electrode surface and enable the sensitive and selective detection of PO.

## 2. Materials and Methods

### 2.1. Reagents

Paraoxon, monocrotophos, *p*-nitrophenol, isocarbophos, and parathion were purchased from Dr. Ehrenstorfer GmbH (Augsburg, Germany). An individual stock solution of pesticide was prepared by dissolving each compound in ethanol with a concentration of 0.1 M of both solutions and stored in amber bottles at 4 °C. *p*-aminothiophenol was obtained from Alfa Aesar (Heifelier, MA, USA). HAuCl_4_·4H_2_O (Au% > 47.8%) was purchased from Sinophacm Chemical Reagent Co., Ltd (Shanghai, China). The other reagents used were commercially available as analytical reagent grade or better.

### 2.2. Apparatus

Disposable SPCEs were purchased from Dropsens, Inc. (Asturias, Spain), which consisted of an Ag/AgCl reference electrode, a carbon working electrode (3 mm in diameter), and a carbon auxiliary electrode. A sensor connector (Alderon Biosciences, Beaufort, NC, USA) allowed for the connecting of the SPCE to a Bio-analytical System (BAS, West Lafayette, IN, USA) CV-50w electrochemical workstation. The scanning electron microscopy (SEM) images were obtained by using a JEOL-6700F SEM instrument (JEOL, Tokyo, Japan). UV spectra were recorded using a UV-2501 spectrophotometer (Shimadzu, Kyoto, Japan).

### 2.3. Preparation of the AuNPs Inner Layer Modified SPCE

A SPCE was first applied at a potential of +1.2 V in acetate buffer (pH 5.0) for 300 s, and then cycle scanned from +0.3 V to +1.1 V at a rate of 50 mV·s^−1^ until a steady current–voltage curve was obtained. The electrode was further washed three times with distilled water and then dried under nitrogen gas. To electrodeposit AuNPs on the SPCE surface, the electrode was applied at a potential of −0.2 V for 80 s in 0.2 g·L^−1^ HAuCl_4_ solution. Optimization of the deposition parameters can be seen in Section 1.1 and Figure S1 in Supplementary Materials. After being cleaned with ethanol and doubly distilled water, the AuNPs/SPCE was immersed in a fresh piranha solution (H_2_SO_4_/H_2_O_2_, 7:3) for about 300 s, rinsed again with doubly distilled water, and finally dried under nitrogen gas. 

### 2.4. ATP Modification and PO Self-Assembly on the AuNPs Inner Layer

ATP modification was carried out by immersing the pretreated AuNPs/SPCE into a 10 mM solution of ATP in ethanol. After reacting at RT for 24 h, the AuNPs/SPCE was taken out and then rinsed three times with ethanol and distilled water, respectively. Afterwards, the AuNPs/SPCE was immersed into a PO solution (1 mM) for 6 h, and then was taken out, rinsed with ethanol, and subsequently dried under nitrogen flow at RT.

### 2.5. Preparation of the Imprinted PATP/AuNPs/SPCE

The pretreated PO–ATP–AuNPs/SPCE was immersed in the HAc–NaAc (pH 5.0) aqueous electrolyte solution containing 5 mM ATP and 0.1 mM PO. The electropolymerization was performed by seven cyclic scanning from −0.20 V to +0.60 V at a scan rate of 50 mV·s^−1^. Optimization of the scan parameters on the PATP film formation can be seen in Section 1.2 in Supplementary Materials. Then, the imprinted PATP/AuNPs/SPCE was rinsed with ethanol three times for 5 min, and then potentially cycled between +0.60 and −1.00V in acetate buffer solution for three cycles to remove the PO template [25]. The complete removal of the template molecules was confirmed by the disappearances of the characteristic differential pulse voltammetric (DPV) response and the UV absorption of PO in the extracting solution. Finally, the imprinted PATP/AuNPs/SPCE was rinsed with distilled water and ethanol, respectively, and subsequently dried under nitrogen for further use. The nonimprinted control was prepared and treated in the same way, except with omit PO.

### 2.6. Electrochemical Measurements

The modified electrodes were allowed to pre-absorb PO by incubating in the acetate buffer solution containing varying PO concentrations for 10 min. After removing the physically adsorbed substances on the electrode surface by doubly distilled water rising, 20 µL of acetate buffer solution was placed onto the working electrode. The amount of bound probe was quantified by DPV measurements from −0.50 V to −1.00 V. The corresponding maximum current was used for concentration determination. All the measurements were performed at RT.

### 2.7. Real Sample Preparation

Apple and cabbage samples were purchased from a local market at Wuhan, China. The real samples were prepared by peeling and chopping the fruit and vegetables into small pieces. Twenty-five grams of the homogenized sample was mixed with 50 mL of acetonitrile and subsequently blended for 3 min. The extract was then filtered through a filter paper into an evaporation flask with 5–7 g of sodium chloride. The mixture was blended for 1 min and let stand for 30 min to let the phases separate. After phase separation, 10 mL of the upper acetonitrile layer was drained and filtered through a 0.2 μm pore size membrane into an evaporation flask, and then evaporated to dryness, following which ethanol (5 mL) was added. Spiking of the samples was achieved by adding different levels of PO stock solution into the samples, and then homogenizing the mixtures.

## 3. Results and Discussion

### 3.1. Preparation of the Imprinted PATP/AuNPs Composite Film

In this work, we aim to enhance the sensitivity and selectivity of PO analysis by modifying the electrode surface with electron-rich amino groups, as well as π-donor aromatic ring groups that would selectively concentrate PO at the electrode surface using hydrogen bonds and π-donor-acceptor interactions. ATP is an appropriate molecular anchor for anchoring template molecules on Au surfaces. Thus, ATP was chosen to act as the polymerizable functional monomer for preparing the PO imprinted polymer films. Furthermore, AuNPs were introduced onto the sensor surface, serving as a high conductive nanostructured imprinted substrate that has a higher surface area, for further increasing the amount of effective imprinted sites [26]. The preparation procedures of the imprinted PATP/AuNPs/SPCE are displayed in Scheme 1a.

In the first procedure, AuNPs were easily formed in situ on the surface of SPCE by electrochemical deposition of HAuCl_4_ at a constant potential of −0.20 V for 80 s. Then the AuNPs-modified SPCE was immersed into the ATP solution for 24 h. The ATP molecules could absorb onto the AuNPs surface based on the Au–S bonds and the exposed an array of the amino groups towards the solution, forming an ATP SAM [27] (see Scheme 1b). The ATP–AuNPs can serve as an initial matrix to develop the surface-imprinted nanoparticles. Meanwhile, the polymerizable ATP monolayer is expected to improve the “wetting” of the AuNPs/SPCE surface by the functional monomer, which may drive the selective occurrence of polymerization at the electrode surface, forming the uniform and the compact polymer film [24].

The imprinting procedures of PO can be seen in step ii and iii of Scheme 1a. Immersing the ATP–AuNPs/SPCE into a 1 mM PO solution for another 6 h allowed for the assembly of PO molecules onto the ATP monolayer through the π–donor–acceptor interactions, as well as hydrogen bond interactions between ATP and PO, forming orderly surface imprinted sites. Then these PO molecules were embedded into the imprinted PATP film during the following electropolymerization process in present of additional PO, thus the imprinted sites were further replenished.

### 3.2. Spectroscopic, Electrochemical, and Microscopic Characterizations

**Figure 1:**
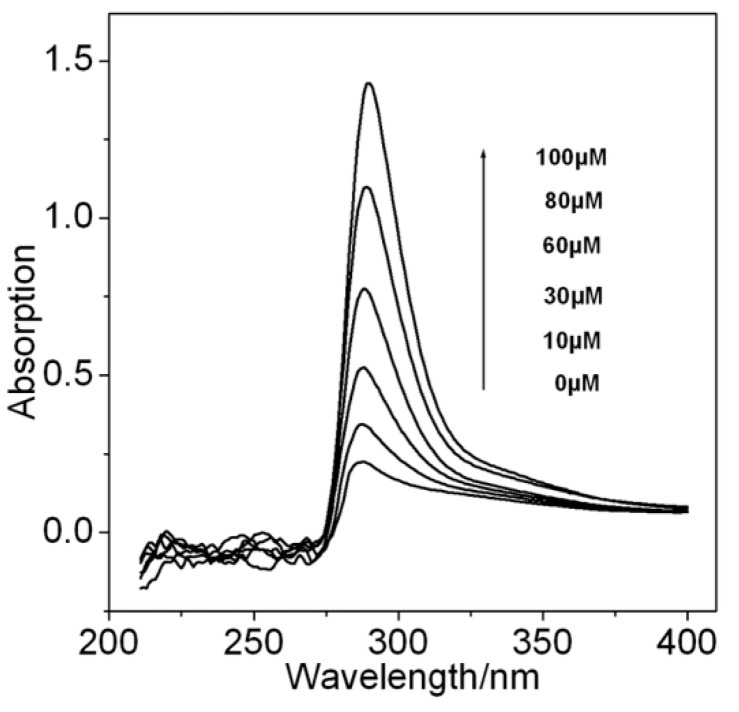
Evolution of UV spectra with increasing different amounts of paraoxon into 10 µM ATP solution.

The intermolecular interaction between ATP and PO was investigated by the measurement of the UV spectra. With the addition of PO into an ATP solution, the maximum absorption wavelengths of ATP slightly shifted to red wavelength form 285 to 289 nm, and strengthened continuously with the increase of the PO amount (see Figure 1). These observations of visible absorption clearly demonstrate that the strong hydrogen bonds and π–donor–acceptor interactions between the ATP and PO molecules, which would drive PO molecules to assemble onto the surface of the ATP monolayer, modified AuNPs/SPCE.

**Figure 2:**
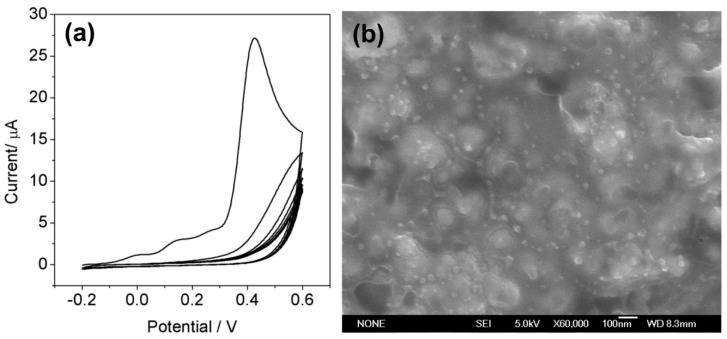
(**a**) Cyclic voltammograms for the electropolymerization of PATP at the ATP–AuNPs/SPCE surface in HAc–NaAc solution (pH 5.0), containing 5 mM ATP and 0.1 mM PO: scan rate, 50 mV·s^−1^; (**b**) Typical scanning electron microscopy (SEM) image of the imprinted PATP/AuNPs film.

Film formation was monitored through the changes in the current per cycle. Figure 2a shows representative CV for the electropolymerization process of ATP on the ATP–AuNPs/SPCE in the presence of PO. At each CV cycle, the oxidation current decreases, suggesting a stepwise growth of the polymer film and resulting in the suppression of the voltammetric response. There is not any difference obtained in the presence/absence of PO from CVs (see Supplementary Materials Figure S2), demonstrating that PO does not exhibit any electrochemical activity in the potential range from −0.2 V to +0.6 V. Therefore, the molecular structure of PO is not affected during electropolymerization. The top morphology of the subsequently formed PO imprinted PATP/AuNPs film was shown in Figure 2b; the imprinted PATP/AuNPs film displayed a uniform and compact morphology, meanwhile, and the visible AuNPs of dimension ca. 12 nm were evenly distributed on the electrode surface.

### 3.3. Sensitivity Enhancement with the Imprinted PATP/AuNPs/SPCE

**Figure 3:**
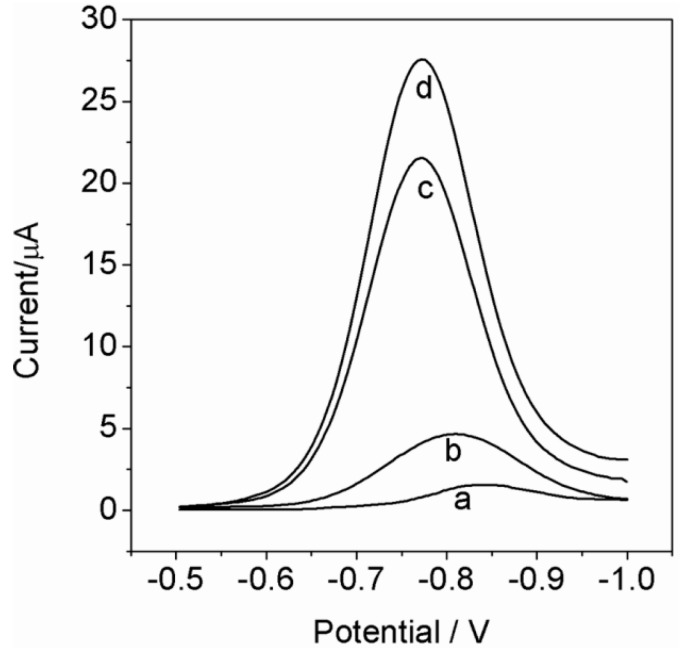
Differential pulse voltammetric (DPV) responses of the (**a**) bare SPCE, (**b**) imprinted PATP/SPCE, (**c**) conventional one-step electropolymerized imprinted PATP/AuNPs/SPCE, and (**d**) self-assembly directed electropolymerization imprinted PATP/AuNPs/SPCE in the presence of 50 μM PO in HAc–NaAc (pH 5.0).

The amperometric response of the developed sensor towards PO was tested at a 50 µM PO solution and compared to the conventional one-step electropolymerized imprinted PATP/AuNPs/SPCE, the imprinted PATP/SPCE, and the bare SPCE. The conventional one-step electropolymerized imprinted control was prepared by immersing the AuNPs/SPCE into the electrolyte solution containing ATP and PO molecules, and following with an electropolymerization step (as described in Section 2.5). The self-assembling of ATP and PO was omitted from the preparation procedure of the conventional control, as compared to that of the developed sensor. Meanwhile, the imprinted PATP/SPCE was prepared and treated in the same way as the conventional one-step electropolymerized imprinted PATP/AuNPs/SPCE, except that the electrodeposition of AuNPs was omitted. As shown in Figure 3, the DPV response that corresponded to the electrochemical reduction of PO at the bare SPCE (see curve a) showed a well-defined reduction peak at ~−0.82 V. The reduction current increased when the SPCE was modified with the imprinted PATP film (see curve b). This phenomenon was attributed to the concentration of the analyte at the electrode surface by the hydrogen bonds and the π–donor–acceptor interactions with the monolayer modifier. Similarly, the one-step electropolymerized imprinted PATP/AuNPs/SPCE (see curve c) displayed an obvious increase in the electrocatalytic reduction current because of the introduction of the AuNPs, which resulted in an increase of the effective surface area and a promotion of the interfacial electron transfer. Furthermore, we observed a more significant increased reduction current (increased to about 28%, compared with the curve c) at the interface of the developed sensor (see curve d). The surface imprinting procedure for preparing the developed sensor through molecular self-assembly of PO on the ATP monolayer would control templates to locate at the electrode surface, increase the amount of effective imprinted sites, and thus enhance the sensitivity of the electrode. The results revealed that by controlling the imprinted sites to locate at the surface of the AuNPs with the highly specific surface area, and further replenishing the imprinted sites by electropolymerized imprinting, the sensitivity of the imprinted PATP/AuNPs/SPCE can be effectively enhanced.

### 3.4. Performance of the Imprinted PATP/AuNPs/SPCE

#### 3.4.1. Sensitivity and Affinity

**Figure 4:**
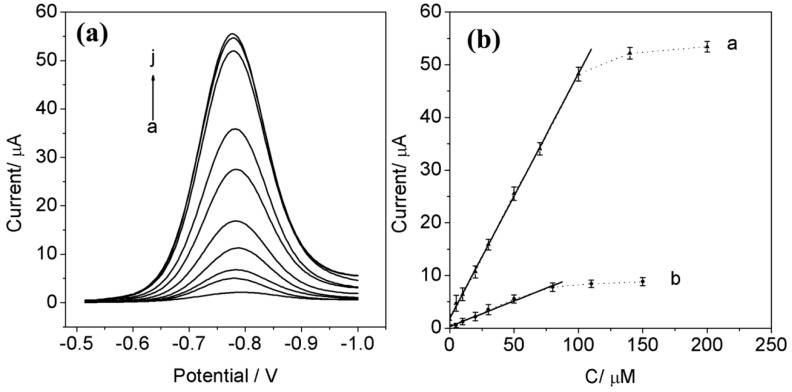
(**a**) DPV responses corresponding to the reduction of PO rebound to the imprinted PATP/AuNPs/SPCE by incubating the sensor in HAc–NaAc solutions (pH 5.0) containing increasing PO concentrations (curves a–j: 1 × 10^−8^ to 2 × 10^−4^ M) for 10 min. (**b**) Calibration curves corresponding to the analysis of PO at the (curve a) imprinted and (curve b) nonimprinted PATP/AuNPs/SPCE.

The DPV responses that correspond to the analysis of PO by the imprinted PATP/AuNPs/SPCE are shown in Figure 4a. Figure 4b shows the linear calibration curve for analyzing PO by the imprinted PATP/AuNPs/SPCE (see curve a) in the PO concentration range from 1 × 10^−8^ to 1 × 10^−4^ M, with a correlation coefficient of 0.9992. The limit of detection (LOD) was determined using the criterion *S/N* = 3 was 1 × 10^−9^ M. For comparison, curve b depicts the calibration curve observed with the nonimprinted PATP/AuNPs/SPCE. The LOD for analyzing PO by the nonimprinted PATP/AuNPs/SPCE is 1.7 × 10^−7^ M. The results show that the LOD for PO by the imprinted PATP/AuNPs/SPCE is about two orders of magnitude lower than that with the nonimprinted control.

**Figure 5:**
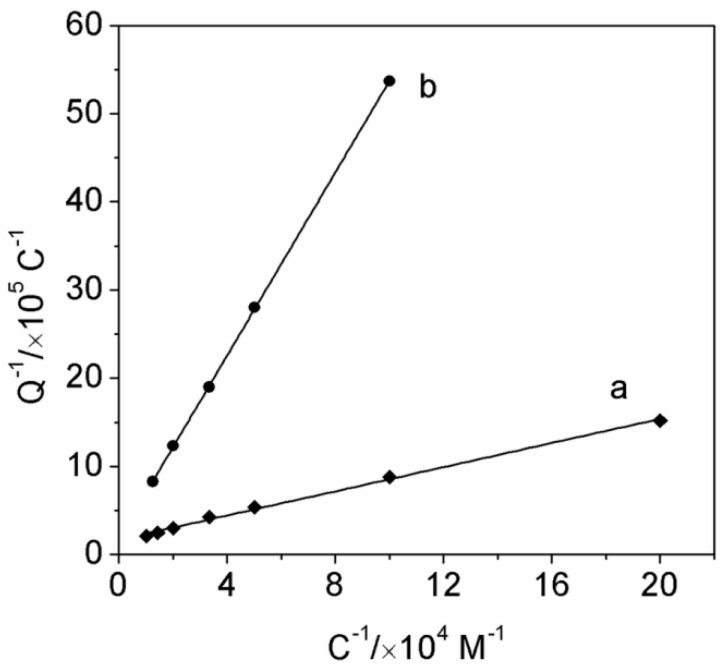
Coulometric analysis of the PO associated with the (**a**) imprinted and (**b**) nonimprinted PATP/AuNPs/SPCE upon interaction of the electrode with different bulk concentrations of PO for 10 min.

To account for the enhanced sensitivity observed with the imprinted PATP/AuNPs/SPCE, we analyzed the association constant (*K*_α_) of PO to the imprinted sensing surface on the basis of Equation (1) [28]. *K*_α_ is given by Equation (2), where α is defined as the amount of imprinted sites in the system, and θ is defined as the amount of imprinted sites that are occupied by PO, which can be derived from the coulometric analysis of the first wave of the reduction of PO at any concentration. Equation (2) can also be rearranged to form Equation (3). The charge associated with the bound PO is proportional to the amount of occupied, imprinted sites. Curve a in Figure 5 shows the coulometric analysis of the PO bound to the imprinted PATP/AuNPs/SPCE at different bulk concentrations of PO, according to equation 3. The derived *K*_α_ with the imprinted PATP/AuNPs corresponded to (2.5 × 10^4^ M^−1^), that is, ca. 7-fold higher than that with the nonimprinted control (*K*_α_ = 3.5 × 10^3^ M^−1^, see curve b in Figure 5). Therefore, the enhanced sensitivity for analyzing PO by the imprinted electrode can be attributed to the improved concentration of PO at the electrode surface, which resulted from the higher affinity of PO to the imprinted sites.

(1) PO+imprintedsites(α−θ)⇌imprintedsites−PO(θ)

(2) $$K_{\alpha }=\frac{\theta }{\left(\alpha -\theta \right)\left[PO\right]}$$

(3) $$\frac{1}{\theta }=\frac{1}{\alpha }+\frac{1}{\alpha \cdot K_{\alpha }\left[PO\right]}$$

#### 3.4.2. Selectivity

**Figure 6:**
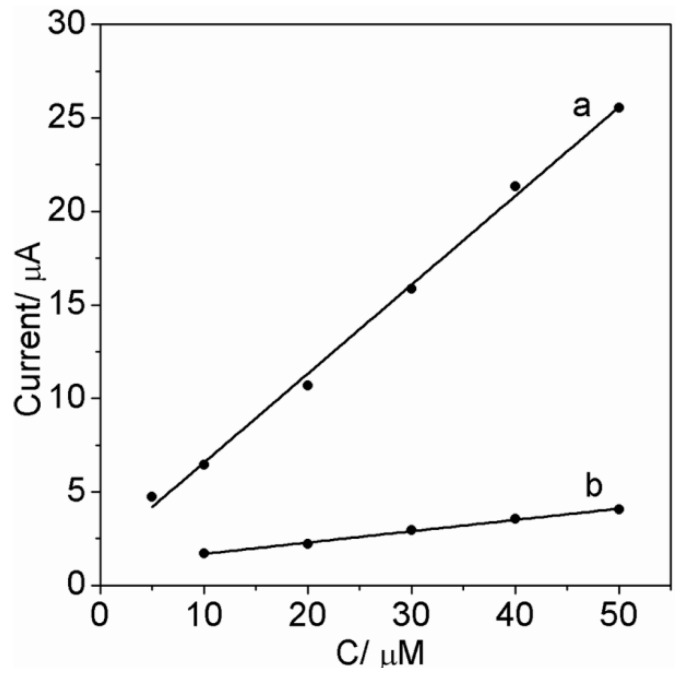
Calibration curves corresponding to the analysis of PO (curve a) and PT (curve b) by the imprinted PATP/AuNPs/SPCE. All data were recorded after interacting the respective electrode with the PO or PT solution sample for 10 min.

We studied the selectivity of the imprinted PATP/AuNPs/SPCE and compared it to the nonimprinted control. Parathion (PT) was used to verify the response selectivity of the imprinted PATP/AuNPs/SPCE towards PO. The response selectivity factor (*k*_PO_/*k*_PT_) of the imprinted PATP/AuNPs/SPCE equals 8 (see Figure 6), where k is defined as the slope of the respective calibration curve. The selectivity of the imprinted PATP/AuNPs/SPCE is ca. 7-fold higher than the nonimprinted control, which has a PO response selectivity factor value 1.1 (see Supplementary Materials Figure S3).

The competitive selectivity property to PO of the developed sensor was evaluated by testing its DPV responses in the presence of some possible interfering substances, including PT, monocrotophos (MC), isocarbophos (IC), and *p*-nitrophenol (*p*-NP), respectively. The competitive selectivity of the imprinted PATP/AuNPs/SPCE electrode to PO molecules was evaluated by calculating the peak current ratio (*I*_p_/*I*_a_), where *I*_p_ and *I*_a_ were the anodic peak current of 10 µM PO at −0.77 V in the presence and absence of interfering substances. The results showed a 50-fold excess of PT, MC, IC, and *p*-NP over PO hardly causes the significant change of peak current of PO, in which the peak current ratio only slightly varied from 0.93 to 1.06 (see Supplementary Materials Table S1), which indicated that the imprinted PATP/AuNPs/SPCE showed higher recognition selectivity for PO than for other structurally similar chemicals. Therefore, the imprinting procedure not only increases the sensitivity of the modified electrode, but also enhances its selectivity towards PO.

### 3.5. Reproducibility and Stability

The reproducibility of the developed imprinted sensor was determined by comparing the DPV responses of ten sensors prepared in parallel under the same conditions towards 10 µM PO in acetate buffer solutions. A relative standard deviation (RSD) of 1.53% was obtained, indicating a good reproducibility in the sensor fabrication. When not in use, the sensor can be put in a plastic casket filled with nitrogen and stored at 4 °C. No obvious deterioration in the response of the sensor towards PO was determined in the first 10-day storage. After a month in storage, the electrode showing a ca. 10% decrease in the PO signal, and a 16% decrease after three months in storage. The obtained results indicate that the developed sensor possesses excellent stability.

### 3.6. Real Sample Analysis

To investigate the feasibility of the imprinted PATP/AuNPs/SPCE for practical applications, apple and cabbage samples were used for the quantitative analysis by the developed sensor. None of these real samples had electrochemical responses when analyzed by using the developed imprinted sensor. It is assumed that there is no PO in the samples, or the concentration of PO is too low to be detected. Thus, the recovery experiments were performed by adding known concentrations of PO into the samples. The samples were then spiked with different levels of PO. Table 1 clearly demonstrates that the performance of the imprinted PATP/AuNPs/SPCE for the detection of PO is not greatly affected by PO in different samples. The average recoveries range between 95.2% and 103.2%, with less than 3.1% RSD (*n* = 6). The presented results demonstrate that the proposed method can be a promising approach for PO determination in real samples. The proposed sensor was compared with other published methods [29,30,31,32,33,34,35,36,37,38] and the data is shown in Table 2. The results reveal that the proposed sensor in this work possesses competitive advantages over other reported sensors, especially the broader linear range with a high fitting degree. The boarder linear range performance of the imprinted film demonstrates it has a high binding capacity towards PO, which is one of the biggest concerns for a PO sensor, and it exhibits its superiority in detecting PO.

## 4. Conclusions

A novel molecularly imprinting strategy, combining surface molecularly imprinting and self-assembly directed electro-polymerization for the sensitive electrochemical sensing of PO was developed. The proposed method relies on the π–donor–acceptor and hydrogen bond interactions between PO, ATP, and ATP–AuNPs. The tailor-made cavities formed in the imprinted film showed good selectivity and affinity toward PO. The selectivity, reproducibility, and stability of the MIP sensor are satisfactory. The good sensitivity, affinity, and selectivity of the sensor is attributed to the following reasons: on the one hand, the employment of the AuNPs inner layer as a high-conductive, nanostructured, imprinted substrate enlarges the electro-active surface area; on the other hand, the surface imprinting of the template and the functional, monomer, self-assembly directed electro-polymerization increases the quantity of the template molecule in the unit area, and creates the imprinted materials with a high ratio of effective imprinted sites. Therefore, this novel, facile strategy reported herein can be further expected to fabricate various molecular imprinted polymers for detecting the pesticide residuals and other environmentally deleterious chemicals.

## Figures and Tables

**Table 1:** The determination results of PO in real samples (*n* = 6).

Sample	Spiked/μM	Found/μM	Recovery (%)	RSD (%)
Apple	0.5	0.487	97.4	1.7
	1.0	0.963	96.3	2.2
	5.0	5.162	103.2	2.8
Cabbage	*0.5*	0.476	95.2	2.3
	*1.0*	1.024	102.4	2.6
	*5.0*	4.932	98.6	3.1

**Table 2:** Performance comparison of the imprinted PATP/AuNPs/SPCE sensor in this work and other reported sensors for determining PO.

Sensor	Linearity (M)	LOD (M)	Ref.
AChE/Au NPs–MWNTs/GCE	1 × 10^−10^–7 × 10^−9^	1 × 10^−9^	[29]
AChE/CPE	3 × 10^−9^–8.3 × 10^−9^	3 × 10^−9^	[30]
AChE/PB/SPCE	5 × 10^−8^–6.3 × 10^−5^	3.6 × 10^−8^	[31]
BuChE/PBNPs/SPCE	7 × 10^−9^–3.6 × 10^−8^	3.6 × 10^−9^	[32]
OPH/micro-ITIES/PDMS	5×10^−7^–1 × 10^−4^	5 × 10^−7^	[33]
OPH/SWNTs/GCE	5 × 10^−7^–8.5 × 10^−6^	1 × 10^−8^	[34]
OPH/CPE	2 × 10^−8^–1.8 × 10^−7^	2 × 10^−8^	[35]
Pt and Ir NPs/BDD	−	2 × 10^−7^	[36]
Bi/Gr/GCE	5 × 10^−9^–4.0 × 10^−8^	2 × 10^−9^	[37]
MIP/CPE	3.8 × 10^−9^–7.5 × 10^−7^	1.0 × 10^−9^	[38]
sensor in this work	1 × 10^−8^–1 × 10^-4^	1 × 10^−9^	

AChE-Acetylcholinesterase; M(S)WNTs—Multiwalled (Single-walled) carbon nanotubes; GCE-Glassy carbon electrode; CPE–Carbon paste electrode; PB–Prussian blue; BuChE–Butyrylcholinesterase; ITIES-Immiscible liquid/liquid electrolyte solutions; OPH–Organophosphorus hydrolase; PDMS–polydimethylsiloxane; BDD–Boron doped diamond.

## Supplementary Materials

Supplementary materials are available online at www.mdpi.com/2073-4360/9/8/359/s1. A pdf file containing the following: Section 1. Optimization of parameters for preparing the imprinted PATP/AuNPs/SPCE; Figure S1: SEM images of AuNPs deposited at different potentials; Figure S2. Cyclic voltammograms for the electropolymerization of PATP at the ATP-modified AuNPs–SPCE surface in HAc-NaAc (pH 5.0) solution containing 5 mM ATP; Figure S3. Calibration curves corresponding to the analysis of (a) PO and (b) PT by the imprinted PATP/AuNPs/SPCE; Table S1. Competitive selectivity of PO imprinted PATP/AuNPs/SPCE toward several structurally similar interferentials.
