# Supplementary material for: Surface Molecularly Imprinted Polymer Film with Poly(p-aminothiophenol) Outer Layer Coated on Gold Nanoparticles Inner Layer for Highly Sensitive and Selective Sensing Paraoxon
Source: Polymers (Basel). 2017 Aug 12;9(8):359. doi: 10.3390/polym9080359 (PMC6418602; doi:10.3390/polym9080359)
Supplement: Supplementary file 1 [file polymers-09-00359-s001.pdf]

Supplementary Materials: Surface Molecularly Imprinted Polymer Film with Poly(*p*-aminothiophenol) Outer Layer Coated on Gold Nanoparticles Inner Layer for Highly Sensitive and Selective Sensing Paraoxon

1 Optimization of parameters for preparing the imprinted PATP/AuNPs/SPCE

1.1 Optimization of the deposition parameters on AuNPs deposition

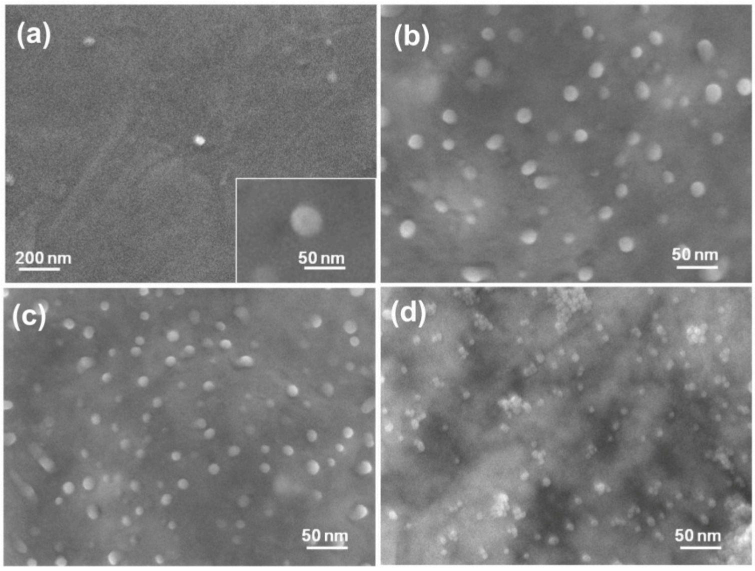

Figure S1. SEM images of AuNPs deposited at different potentials: **(a)** 0.2 V, **(b)** 0.0 V, **(c)** −0.2 V, **(d)** −0.4 V.

The nucleation potential and deposition time was used to control the size and the amount of the deposited AuNPs, respectively, for obtaining an optimum coverage of AuNPs on the electrode surface. The effect of deposition potential on particle size and density for a constant charge passed (6×10^{-4} C) has been investigated. It is clear from the images (see Figure S1) that the size of the AuNPs decreased and the density of the Au NPs increased with decreasing electrode potential from 0.2 to −0.4 V vs. Ag/AgCl while keeping the total coverage in terms of Au atoms constant on the surface by controlling the amount of charge deposited. It may be due to the low overpotential (0.2 V) resulted in a lower density of nucleation sites and leaded to large and sparsely dispersed AuNPs [1]. While the higher overpotentials (0.0 and −0.2V) are energetically favorable the formation of larger numbers of nucleation sites all over the electrode surface, hence resulted in the smaller and densely dispersed AuNPs. However, we can clear see that small agglomeration of AuNPs occurred when a high deposited overpotential (−0.4 V) was used, which may due to the increased amount of nucleation sites as well as high grow rates of AuNPs. Therefore −0.2 V vs. Ag/AgCl was selected as the optimal potential for AuNPs deposition.

At this optimized potential, the ideal deposition time for AuNPs was also determined by measuring the voltammetric response of corresponding electrodes to Fe $(CN)_6^{3-/4-}$ probe, and got the ideal deposition time 80s. Although the longer deposition time could increase the amount of AuNPs on the electrode surface, it can induce aggregations of AuNPs on the

electrode surface and resulting a decrease of the surface area of AuNPs, leading to a decrease in voltammetric response of Fe $(CN)_6^{3-/4-}$. Under the optimum condition (potential: −0.2 V vs. Ag/AgCl, time: 80 s) the average diameter and surface coverage of the Au NPs formed on the electrode surface was analyzed on the basis of SEM images. The obtained values that corresponds to the AuNPs diameter and surface coverage were 10~15 nm and 2.5×10^{11} Au NPs per cm^{-2}, respectively.

1.2. Optimization of the deposition parameters on PATP film formation.

It is well known one major issue with the surface imprinting is controlling the imprinted polymer film thickness for providing rapid mass transfer [2]. Too thick imprinted polymer films would induce incompletely removal of template molecules and exhibit low binding capacity, poor site accessibility, and slow binding kinetics, therefore, the formation of thinner polymer films is preferred for rapid mass transfer. Herein, we optimized the thickness of the PATP film by testing the peak current of PO reduction at the imprinted PATP/AuNPs/SPCE with different scanning cycles during the imprinting electropolymerization process. The DPV response to PO obviously rises and reaches a maximum with the 7 cycles and then decreases with a further increase of cycle number. Therefore, seven cycles can obtain a suitable film thickness to provide the highest sensitivity to PO analyte.

2. Other figures and table

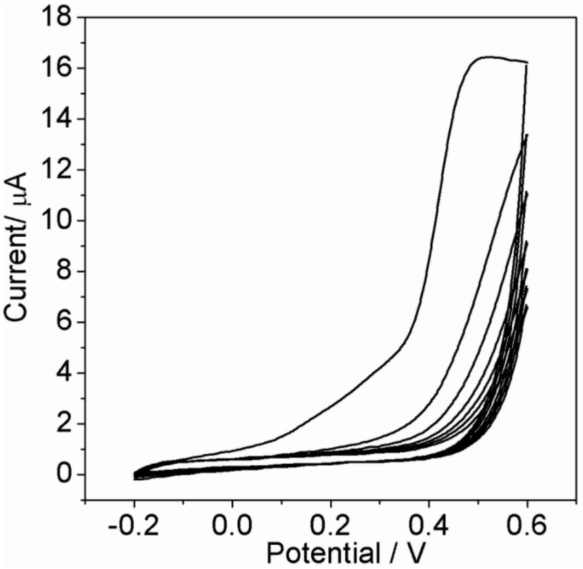

Figure S2. Cyclic voltammograms for the electropolymerization of PATP at the ATP-modified AuNP-SPCE surface in HAc-NaAc (pH 5.0) solution containing 5 mM ATP: scan rate, 50 mV·s^{-1}.

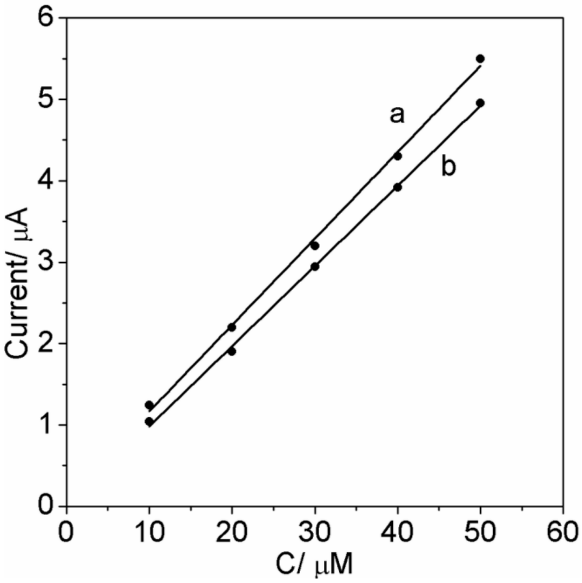

Figure S3. Calibration curves corresponding to the analysis of (a) PO and (b) PT by the imprinted PATP/AuNPs/SPCE. All data were recorded after interacting the respective electrodes with the PO or PT solution sample for 10 min.

Table S1. Competitive selectivity of PO imprinted PATP/AuNPs/SPCE toward several structurally similar interferent[a]

Analyte	Interferent	$(I_p/I_a)^b$
PO	PT	1.03
	MC	0.96
	IC	0.93
	p-NP	1.06

[a] The PO imprinted PATP/AuNPs/SPCE was incubated in acetate buffer solution containing 10 μM PO and 0.5 mM interferent for 10 min; [b] I_p and I_a present DPV responses of PO at −0.77 V in the present and absence of interfering substances.

References

1. Komsiyska, L.; Staikov, G. Electrocrystallization of Au nanoparticles on glassy carbon from HClO4 solution containing $[AuCl_4]^-$.*Electrochim. Acta*. **2008**,*54*,168–172.

2. Gao, D.; Zhang, Z.; Wu, M.; Xie, C.; Guan, G.; Wang, D. A surface functional monomer-directing strategy for highly dense imprinting of TNT at surface of silica nanoparticles. *J. Am. Chem. Soc.* **2007**, *129,* 7859–7866.
